# Dielectric Metalens for Superoscillatory Focusing Based on High-Order Angular Bessel Function

**DOI:** 10.3390/nano12193485

**Published:** 2022-10-05

**Authors:** Yu Li, Xinhao Fan, Yunfeng Huang, Xuyue Guo, Liang Zhou, Peng Li, Jianlin Zhao

**Affiliations:** 1 MOE Key Laboratory of Material Physics and Chemistry under Extraordinary Conditions, and Shaanxi Key Laboratory of Optical Information Technology, School of Physical Science and Technology, Northwestern Polytechnical University, Xi’an 710129, China; 2 Xi’an Ming De Institute of Technology, Xi’an 710124, China

**Keywords:** superoscillation, vector beam, metalens, polarization, diffraction

## Abstract

The phenomenon of optical superoscillation provides an unprecedented way to solve the problem of optical far-field label-free super-resolution imaging. Numerous optical devices that enable superoscillatory focusing were developed based on scalar and vector diffraction theories in the past several years. However, these reported devices are designed according to the half-wave zone method in spatial coordinates. In this paper, we propose a dielectric metalens for superoscillatory focusing based on the diffraction of angular Bessel functional phase modulated vector field, under the inspiration of the tightly autofocusing property of a radially polarized high-order Bessel beam. Based on this kind of metalens with a numerical aperture (NA) of 0.9, the linearly polarized light is converted into a radially polarized one and then focus into a superoscillating focal spot with the size of 0.32*λ*/NA. This angular spectrum modulation theory involved in this paper provides a different way of designing superoscillatory devices.

## 1. Introduction

The development of optical imaging technology has significantly promoted the innovation and technological development of physics, chemistry, materials, biomedicine, and other fields. However, limited by the optical diffraction limit, namely, Abbe’s diffraction limit [1], it is difficult for conventional optical imaging systems to achieve sub-diffractive resolution in these applications. In recent years, many milestone super-resolution techniques have emerged [2,3,4,5,6,7,8], such as fluorescence microscopy [6] and near-field scanning imaging technologies [2], to overcome the critical limit and improve the imaging resolution of optical systems. Whereas, for far-field label-free optical imaging, the imaging resolution is still a challenge to further improve [9]. The discovery of the optical superoscillation phenomenon opens up a new avenue to overcome this problem [10].

Optical superoscillation refers to the phenomenon that the coherent superposition of light fields with lower spatial frequencies form a structured light field in which the local oscillation frequency is greater than its maximum frequency [11,12]. Since the local spatial frequency is greater than the maximum frequency of the system [13], superoscillating light fields can produce a focal spot with a subdiffraction limit scale in the far-field local area without relying on evanescent wave extraction and fluorescent labeling [14,15,16]. In 2006, Berry and Popescu firstly proposed that light field diffracted through subwavelength grating can generate arbitrarily small spatial energy regions without relying on evanescent waves, which theoretically proved the feasibility of using superoscillation methods to improve the resolution of imaging systems [10]. Lately, Zheludev first experimentally demonstrated this typical phenomenon by observing a focal spot of size 0.44*λ* generated from the diffraction of a quasi-periodic metal nano-hole array screen [17]. Subsequently, various devices and design methods for generating superoscillating light fields have been continuously proposed [18,19,20,21,22,23,24,25].

Recently, the rapidly developed metasurfaces, which are made of 2D arrays with anisotropic units and enable the simultaneous modulation of multiple parameters on a subwavelength scale [26,27], bring new opportunities for the development of superoscillatory focusing devices [16,28,29,30,31,32,33,34]. For example, Qin et al. proposed a metalens with binary amplitude modulation capacity, which can generate a needle spot with a length of 12*λ* and size of 0.42*λ* for the incidence of azimuthally polarized light field carrying vortex phase [35]. In 2019, Yuan et al. produced a plasmonic metalens with an effective numerical aperture (NA) of 1.52, which can create a superoscillating hotspot with a size of 0.33*λ* in free space [36].

In this paper, we propose a type of superoscillatory focusing metalens based on the diffraction characteristics of angular Bessel functional phase modulated vector field. By utilizing the tightly autofocusing property of radially polarized high-order Bessel beam [37,38], as well as the Richard–Wolf vector diffraction theory, a dielectric metalens with the capacity of simultaneous modulation of polarization and phase is designed, to create superoscillating focal field with a size that is considerably smaller than the diffraction limit. As a theoretical prediction and experimental demonstration, dielectric metalenses with NA = 0.9, as well as hotspots as small as 0.32*λ*/NA in size, are reported.

## 2. Theory and Method

**Figure 1:**
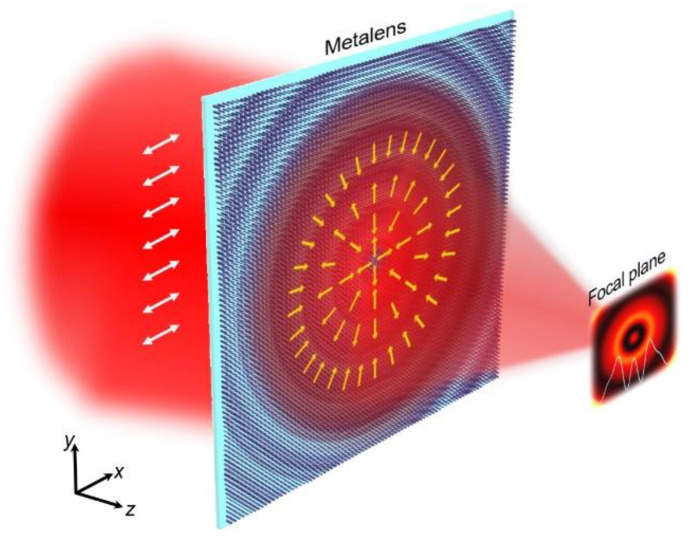
Schematic illustration of the superoscillatory focusing metalens. The arrows depict the instantaneous directions of electric component of the light field input and output from the metalens.

The generation principle of the superoscillating light field is shown in Figure 1. As shown, a linearly polarized beam is incident on the metalens that enables the polarization conversion and phase modulation effects. The transmitted light field is converted into a radially polarized one, whose local polarization direction changes along the radial and azimuthal directions simultaneously, as the yellow arrows show in Figure 1, and then is focused into a focal field with a superoscillatory hotspot.

To design the metalens, we take the advantage of the tightly autofocusing property of higher-order vector Bessel beams. First, considering that the cylindrical vector light field can break the optical diffraction limit under the tightly focusing condition [39,40], the radially polarized vector beam is used as the incident beam. Second, in order to further reduce the size of the focal spot, a phase-type diffractive optical element is introduced to control the wavefront of the tightly focusing vector beam [41,42,43]. Since the incident beam is a fundamental-order one, the modulation phase of the diffractive optical element is designed to be a high-order angular Bessel function, which can be written as:

(1) $$\Phi_{0}\left(\theta \right)=\left\{1+\mathrm{sgn}\left[J_{10}\left(k_{r}f\tan\left(\theta \right)\right)\right]\right\}\pi /2,$$

where *J*_10_(·) is the 10th order Bessel function of the first kind; sgn(·) denotes the sign function; *k_r_* is the transverse wave number; *f* is the focal length of the tightly focusing lens; and *θ* is the discrete opening angle in the focusing model. Different from the traditional half-band principle that has uniform width [12], the width of the half-band here is dependent on the angular function of Equation (1). 

To select the *k_r_*, we numerically calculated the focal field according to the Richards–Wolf vectorial diffraction integral [44,45], whose electric components are expressed as:

(2) $$\left[\begin{array}{c} E_{x}\\ E_{y}\\ E_{z} \end{array}\right]=-\frac{ik_{0}f}{2\pi }\int_{0}^{\theta_{\max}^{}}\int_{0}^{2\pi }A\left(\theta ,\varphi \right)\sqrt{\cos\theta }P\left(\theta ,\varphi \right)e^{ik_{0}\left[r\sin\theta \cos\left(\varphi -\phi \right)+z\cos\theta \right]}\sin\theta d\theta d\varphi ,$$

where the polarization vector **P**(*θ*,*φ*) is:

(3) $$P=\left[\begin{array}{c} a\left[1+\cos^{2}\varphi \left(\cos\theta -1\right)\right]+b\left[\sin\varphi \cos\varphi \left(\cos\theta -1\right)\right]\\ a\left[\sin\varphi \cos\varphi \left(\cos\theta -1\right)\right]+b\left[1+\sin^{2}\varphi \left(\cos\theta -1\right)\right]\\ a\left(-\sin\theta \cos\varphi \right)+b\left(-\sin\theta \sin\varphi \right) \end{array}\right],$$

**Figure 2:**
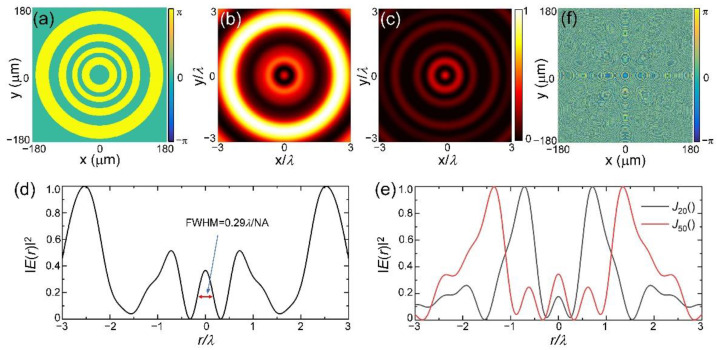
Generation of superoscillatory focal field from the tightly focusing of vector light field. (**a**) Binary phase of the diffractive optical element designed from angular Bessel function with *k_r_* = 0.43*k* and NA = 0.9; (**b**) Simulated total intensity distribution of the focal field; (**c**) The intensity distribution of the longitudinal component *I_z_* at the focal plane; (**d**) Intensity distribution along the radial direction. (**e**) Intensity distributions of focal fields corresponding to the metalens designed from 20th and 50th order Bessel functions of the first kind with *k_r_* = 0.68*k*_0_ and 0.66*k*_0_, respectively; (**f**) Modulation phase of the metalens generated from the combination of tightly focusing phase and binary phase in 2a.

Here, (*r*,*ϕ*,*z*) are the cylindrical coordinates in the focal region, sin*θ*_max_ = NA, *A*(*θ*,*φ*) corresponds to the uniform complex amplitude of input beam under the modulation of phase *Φ*_0_(*θ*), *a* = cos(*φ*) and *b* = sin(*φ*) are the *x*- and *y*-polarized components corresponding to the radial vector beams. Figure 2a shows the modulation phase of such a diffractive optical element when *k_r_* = 0.43*k*_0_ with *k*_0_ = 2π/*λ* the wave number, and the interval of *θ* is 0.1 radian. Figure 2b and 2c exhibit the numerically calculated intensity distributions of the total field and its longitudinal component by using the Richards–Wolf vector diffraction theory. The wavelength is 633 nm and the NA of the focusing lens is 0.9. Figure 2d shows the intensity distribution of the total light field along the radial direction. Clearly, the full width at half maximum (FWHM) of the central hotspot is about 0.29*λ*/NA, which is significantly smaller than the criterion of 0.38*λ*/NA [12], indicating the superoscillation phenomenon. Figure 2e illustrates the intensity distributions of focal fields corresponding to the metalens designed from 20th and 50th order Bessel functions of the first kind with *k_r_* = 0.68*k*_0_ and 0.66*k*_0_, respectively. It can be seen that for high-order Bessel functions, superoscillation can be generated by finding a suitable *k_r_*. Here, we use the 10th-order Bessel function with a different *k_r_* to demonstrate our approach.

Considering the integratable and compact advantages of the metasurface, we assemble multiple functions of polarization conversion, phase modulation of the diffractive optical element, and tightly focusing to design the metalens. Because of the independence of phase and polarization modulations, we first integrate the diffractive optical element with the tightly focusing lens, of which the phase distribution is expressed as *Φ*(*r*) = −*k*_0_($\sqrt{r^{2}+f^{2}}$− *f*), and then generate the combined modulation phase of the metalens, whose profile is shown in Figure 2f. In order to realize polarization modulation, we select geometries whose phase retardation of two orthogonal eigenstates is π, namely, half-wave retardant meta-atoms, as the meta-atom, under the premise of the incidence of linear polarization. For the case of horizontally polarized incidence, the metalens thus can be equivalent to a half-order *q*-plate with a rotation angle of local meta-atom characterized as *α* = *φ*/2.

**Figure 3:**
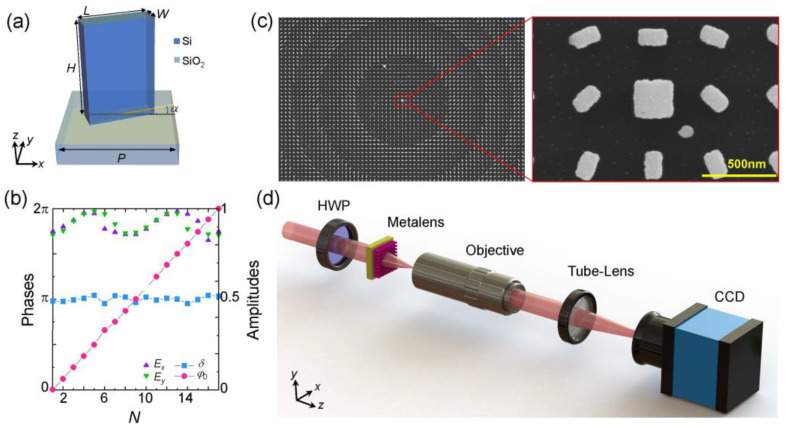
Design and characterization of the metalens. (**a**) Schematic illustration of an element consisting of a poly-Si nanopillar and glass substrate. The geometric parameters of the element are denoted as *H* (height), *L* (length), *W* (width), and *P* (period), the rotation angle is denoted as *α*; (**b**) Transmission amplitude (*E_x_* and *E_y_*) and phase retardation [*δ* = *φ_x_* − *φ_y_* and *φ*_0_ = (*φ_x_* + *φ_y_*)/2] of eigenstates within 16 selected elements; (**c**) Scanning electron microscope images of the metalens and its local structure. The sample is composed of 800 × 800 elements with a lattice constant of 450 nm along *x*- and *y*-axes. The scale bar is 500 nm; (**d**) Sketch of experimental setup, HWP: half-wave plate.

According to this principle, we chose polycrystalline silicon (poly-Si) and SiO_2_ as high refractive index materials and substrates to fabricate the metalens, whose meta-atom is schematically shown in Figure 3a. As shown, the meta-atom consists of a poly-Si rectangle nanopillar deposited on the glass substrate. The height and period of the nanopillar are *H* = 570 nm and *P* = 450 nm, respectively, and the refractive index is *n* = 3.36329 + 0.01162i. We calculated the response of the meta-atom by using a finite-difference time-domain (Lumerical software, Ansys Canada Ltd., Vancouver, Canada) simulation and selected 16 geometric configurations meeting the polarization and phase modulation conditions, that is, two linearly polarized eigenstates (*E_x_* and *E_y_*) that keep a π phase retardation difference, i.e., *δ* = |*φ_x_* – *φ_y_*| *=* π, while the propagation phase increases linearly in an interval of 2π, i.e., $\varphi_{0}^{n}$ = ($\varphi_{x}^{n}$+ $\varphi_{y}^{n}$)/2 *= n*π/16. Figure 3b depicts the transmission amplitude, propagation phase *φ*_0_, and retardation difference *δ* of these 16 configurations. In addition, for the central singularity, we picked out a configuration with near zeroth transmission amplitudes, i.e., *E_x_* ≈ *E_y_* ≈ 0. Its geometric parameters are *L* = 276 nm and *W* = 234 nm. We fabricated the metalens with a transmission-type configuration by using standard electron-beam lithography and inductively coupled plasma etching [46,47]. Figure 3c shows the scanning electron microscope images of the metalens and its local structure. The sample is composed of 800 × 800 elements with a lattice constant of 450 nm along the *x*- and *y*-axes. The experiment is carried out with the setup shown in Figure 3d. A linearly polarized beam from the He-Ne laser is converted into a horizontal one after passing through the half-wave plate, and then a normal incident into the metalens. The superoscillating focal field is generated at the focal plane of the metalens, we used a microscopic measurement system consisting of a 100× objective lens (Mitutoyo, NA = 0.9), tube-lens, and a CCD camera (DMK, 23U445) to observe the focal field.

## 3. Results

**Figure 4:**
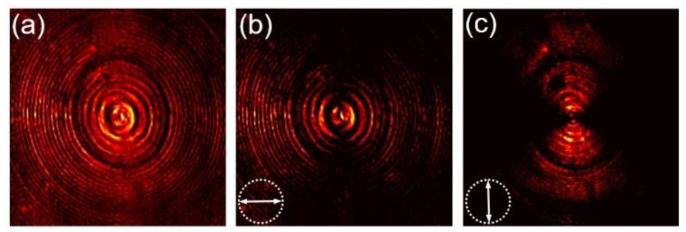
Intensity distribution of light field transmitted from the metalens in the case of horizontally polarized beam incidence. (**a**) Total intensity; (**b**) Horizontal component; (**c**) Vertical component. The arrows depict the orientation of polarization analyzer.

Figure 4 shows the measured intensity distribution near the back face of the metalens, namely, the intensity of the transmitted light field when the metalens is illuminated by a horizontally polarized beam, where these three panels correspond to the total field and its horizontal and vertical polarization components, respectively. As the measured intensity patterns show, the transmitted field presents transverse intensity distribution and variations the same as the radial vector beam, indicating the successful transformation of incident polarization.

**Figure 5:**
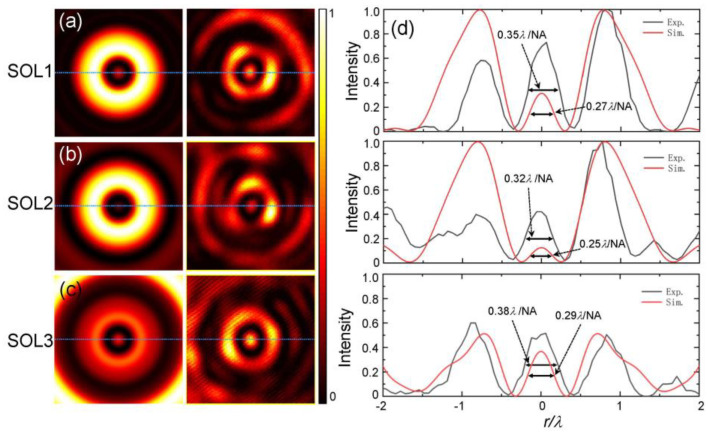
Experimental results of three superoscillatory metalenses. (**a**–**c**) Intensity distributions of the simulated and measured focal fields generated by metalenses with parameters of *k_r_* = 0.08*k*_0_, 0.29*k*_0_, and 0.43*k*_0_; (**d**) Normalized line-scan intensity profiles at the focal plane (indicated by dashed lines): simulation (red) and experiment (black).

In the experiment, we designed three superoscillatory metalenses (named SOL1, SOL2, and SOL3) according to Equation (1), of which the transverse wave vectors are chosen as *k_r_* = 0.08*k*_0_, 0.29*k*_0_, and 0.43*k*_0_, respectively. The simulated and measured intensity distributions of the focal fields are shown in Figure 5a–c. Figure 5d gives the line-scan intensity profiles corresponding to the simulated (red) and measured (black) results indicated by the dashed lines. All results are obtained with the same incident condition and normalized by the maximum intensity. From these simulated results, it can be seen that the central hotspots in three cases both present the superoscillation phenomenon, as shown in Figure 5d. Among them, for the case of *k_r_* = 0.29*k*_0_, the FWHM of the superoscillating hotspot can be reduced to 0.25*λ*/NA. However, the relative intensity of this hotspot with the sideband is the smallest. In this case of *k_r_* = 0.43*k*_0_, although the resulting hotspot has the largest field of view and the lowest relative intensity of the nearest sideband, the size of the central hot spot is the largest, with a magnitude of about 0.29*λ*/NA. These results illustrate that the adopted angular Bessel modulation method has high applicability, and can generate superoscillating focal fields for various requirements by optimizing its parameters. Comparing the theoretical and experimental results, one can find that the experimental results are basically consistent with the theoretical predictions. In practice, the smallest size of the hotspot is about 0.32*λ*/NA when *k_r_* = 0.29*k*_0_, which is still smaller than the superoscillatory criterion of 0.38*λ*/NA, indicating the superoscillatory focusing capability of this type of metalens. In addition, we would like to note that the sizes of the measured focal spots are greater than these theoretical ones. The reason is mainly due to the non-uniform transmission and direct transmission components due to the phase delay errors caused by the fabrication errors and imperfections of the chosen geometry, as shown in Figure 4.

## 4. Conclusions

In conclusion, we proposed a dielectric metalens with independent polarization and phase modulation effects to realize superoscillatory focusing. The metalens was designed based on the tightly autofocusing property of a radially polarized high-order Bessel beam. For the incidence of the linearly polarized beam, it can transform the uniform beam into a radially polarized one with angular Bessel functional phase structures and then produce a superoscillating focal field with a hotspot size as small as 0.32*λ*/NA. Our method offers a different design idea for an optical device for applications such as far-field super-resolution imaging.

## Data Availability

The data presented in this study are available on request from the corresponding author.
